# The use of a commercial vegetable juice as a practical means to increase vegetable intake: a randomized controlled trial

**DOI:** 10.1186/1475-2891-9-38

**Published:** 2010-09-17

**Authors:** Sonia F Shenoy, Alexandra G Kazaks, Roberta R Holt, Hsin Ju Chen, Barbara L Winters, Chor San Khoo, Walker SC Poston, C Keith Haddock, Rebecca S Reeves, John P Foreyt, M Eric Gershwin, Carl L Keen

**Affiliations:** 1 Department of Nutrition, University of California Davis, One Shields Ave, Davis, California 95616, USA; 2 Department of Nutrition and Exercise Science, Bastyr University, Juanita Drive NE, Kenmore, Washington 98028, USA; 3 Global Nutrition Department, Campbell Soup Company, One Campbell Place, Camden, New Jersey 08103, USA; 4 Office of Scientific Affairs, Campbell Soup Company, One Campbell Place, Camden, New Jersey 08103, USA; 5 Institute for Biobehavioral Health Research, National Development and Research Institutes (NDRI), West 143rd Street, Leawood, Kansas 66224, USA; 6 Department of Medicine, Baylor College of Medicine, Travis St, Houston, Texas 77030, USA; 7 Division of Rheumatology, Allergy and Clinical Immunology, University of California Davis School of Medicine, Health Sciences Drive, Davis, California 95616, USA; 8 Department of Internal Medicine, University of California Davis School of Medicine, V Street, Sacramento, California 95817, USA

## Abstract

**Background:**

Recommendations for daily dietary vegetable intake were increased in the 2005 USDA Dietary Guidelines as consumption of a diet rich in vegetables has been associated with lower risk of certain chronic health disorders including cardiovascular disease. However, vegetable consumption in the United States has declined over the past decade; consequently, the gap between dietary recommendations and vegetable intake is widening. The primary aim of this study is to determine if drinking vegetable juice is a practical way to help meet daily dietary recommendations for vegetable intake consistent with the 2005 Dietary Guidelines and the Dietary Approaches to Stop Hypertension (DASH) diet. The secondary aim is to assess the effect of a vegetable juice on measures of cardiovascular health.

**Methods:**

We conducted a 12-week, randomized, controlled, parallel-arm study consisting of 3 groups of free-living, healthy volunteers who participated in study visits at the Ragle Human Nutrition Research Center at the University of California, Davis. All subjects received education on the DASH diet and 0, 8 or 16 fluid ounces of vegetable juice daily. Assessments were completed of daily vegetable servings before and after incorporation of vegetable juice and cardiovascular health parameters including blood pressure.

**Results:**

Without the juice, vegetable intake in all groups was lower than the 2005 Dietary Guidelines and DASH diet recommendations. The consumption of the vegetable juice helped participants reach recommended intake. In general, parameters associated with cardiovascular health did not change over time. However, in the vegetable juice intervention groups, subjects who were pre-hypertensive at the start of the study showed a significant decrease in blood pressure during the 12-week intervention period.

**Conclusion:**

Including 1-2 cups of vegetable juice daily was an effective and acceptable way for healthy adults to close the dietary vegetable gap. Increase in daily vegetable intake was associated with a reduction in blood pressure in subjects who were pre-hypertensive at the start of the trial.

**Trial Registration:**

Clinicaltrials.gov NCT01161706

## Background

For almost two decades, the United States Department of Agriculture (USDA) has recommended that Americans consume a minimum of 5 servings of vegetables and fruits a day [1]. These recommendations are based on numerous studies showing that meeting the recommended daily intake of fruit and vegetables is associated with a reduced risk for a number of chronic diseases, with the evidence for cardiovascular disease and stroke being particularly strong [2-5]. In 2005 the Department of Health and Human Services updated the Dietary Guidelines which recommended an increase in fruit and vegetable intake and changed the recommendations from servings to cups [6]. The new goal was 2-6.5 cups (equivalent to 4 - 13 servings) according to an individual's sex, physical activity and age [6]. For this study, which focuses on vegetable consumption, the Dietary Guidelines suggest that 2½ cups of vegetables per day are recommended for a reference 2,000-calorie intake [6].

Despite numerous intensive public educational campaigns, surveys examining trends in vegetable consumption from 1988 to 2002 show that 7 out of 10 Americans consume less than the USDA recommended servings of vegetables each day and suggests that 25 percent of respondents had no daily vegetable intake at all [7]. There are multiple reasons for low vegetable intake in the United States, including perceived high costs [8], lack of awareness of the benefits of vegetable consumption [9-11], low availability of fresh products in certain areas [12], taste concerns [13], and limited food preparation time and skills [14]. A consequence of low vegetable consumption can be a low intake of essential nutrients that are key factors for vascular health such as potassium and vitamin C, as well as decreased sources of phytochemicals, such as flavonoids and carotenoids, which have a number of putative positive vascular health benefits [15,16]. Given these factors, there is a need to develop new strategies that can close the gap between what is recommended for vegetable intake, versus what is actually occurring in the general population. Logically the solutions that are advanced for closing the gap between intake and recommendations should be convenient, calorie appropriate, cost effective, and supported by sound science [17].

The Dietary Approach to Stop Hypertension (DASH) diet was originally designed for those with cardiovascular risk factors, such as hypertension [18,19], but it was subsequently recommended as one of two healthy eating patterns for the general U.S. population in the 2005 Dietary Guidelines [6]. Adherence to the DASH diet has been shown to be associated with improved vascular health in a number of trials [9,20,21].

The primary objective of the current study was to determine if the incorporation of a vegetable juice is a practical way for individuals to help meet dietary recommendations for daily vegetable intake as described by the 2005 Dietary Guidelines and the DASH diet. Other goals were to obtain preliminary evidence about the impact of vegetable juice consumption on cardiovascular health by measuring blood pressure, lipid profiles and other related parameters.

## Methods

### Study Population

Adult men and women (age 40-65 years) were recruited from the University of California, Davis, campus and surrounding Sacramento, Solano and Yolo counties using newspaper advertisements. One hundred and sixty two individuals were screened at the Ragle Human Nutrition Research Center for the study, of which 90 (66 women and 24 men) were enrolled into the study. The ethnicities of those enrolled were representative of the recruitment area and included: 63% Caucasian, 9% Asian, 10% Hispanic, 5% other, and 13% not reported. Exclusion criteria were: use of medications that affect blood clotting and vascular reactivity such as anti-depressants, anti-anxiety medications, and aspirin, blood pressure ≥ 140 mm Hg systolic or ≥ 90 diastolic, BMI < 18 or ≥ 35 kg/m^2^, uncontrolled diabetes or hypertension, history of heart disease, and a Beck Depression Inventory^® ^(BDI) depression scale score of 21 or above (Pearson Education, Inc., San Antonio, Texas). With the exception of basic multivitamin/mineral supplements, subjects were instructed to refrain from using dietary supplements, including herbs and omega-3 fatty acids during the study period. Participants were instructed to refrain from using nonsteroidal and anti-inflammatory medication for the week prior to a clinic visit. All subjects provided written informed consent at the time of screening, and the Institutional Review Board at the University of California, Davis, approved this study.

### Design

Eligible subjects were randomized in a three-arm parallel design to drink 8 fluid ounces (1 cup) or 16 fluid ounces (2 cups) of vegetable juice or no juice for a 12-week period. Visits were at baseline (week 0), week 6 and week 12 of the study. Subjects were instructed to follow a low carotenoid diet for the week prior to study day 1 so that all subjects started with the same approximate baseline carotenoid level [22] and a low flavonoid diet 24 hours prior to each visit. During the low flavonoid diet, subjects continued to consume their vegetable juice. Previous studies have shown that these dietary phytochemicals can have an impact on vascular function [23-25]. Three day diet records were collected during the low carotenoid diet period to assess adherence to the diet restriction.

All participants were asked to follow the DASH diet plan which emphasizes vegetables, fruits, whole grains, lean meats and low fat or fat free milk [6]. It is rich in magnesium, potassium, calcium and fiber. In line with the USDA Food Guide, the DASH diet defines a serving of vegetables as one cup of raw leafy vegetables or half a cup of vegetable juice, cooked vegetables or raw vegetables [26,27]. In this paper, we report vegetable intake as DASH servings. The DASH diet training emphasized inclusion of vegetables, and specifically did not instruct subjects to consume vegetable juice in place of mealtime vegetable servings. On the first study day all subjects were counseled about the diet in small groups. The curriculum for the study was based on six objectives and during the session participants were expected to:

1. Identify the key aspects of the DASH eating plan.

2. Calculate the appropriate number of servings for each food group according to a personal calorie goal.

3. Describe ways of measuring appropriate serving sizes of foods.

4. Develop realistic personal goals and meal plans.

5. Discuss tips to make healthy eating easier.

6. Demonstrate how to use a checklist to track their individual progress towards meeting the DASH goals.

For each objective, appropriate handouts were provided and filled out during the instruction period and participants were given the material to take home.

Participants randomly assigned to the vegetable juice groups were supplied with vegetable juice to last for the 6 weeks between visits. The juice was packaged in 46-fluid ounces bottles with a plain black and white label. The same lot was used for all subjects for the 12-week study period. A clear plastic glass with an 8 fluid ounce marker was provided for ease of juice measurement. Eight fluid ounces of the vegetable juice (V8^®^; Campbell Soup Company, Camden NJ) provided 50 calories, 0 g of total fat and cholesterol, 480 mg of sodium, 470 mg of potassium, 2 g of protein, 20 mg lycopene, and 10 g of total carbohydrate of which 2 g were dietary fiber, and 8 g of sugars. The juice provided 40% of the Daily Value of Vitamin A from naturally occurring beta-carotene in the vegetables (1000 IUs = 300 micrograms RAEs (Retinol Activity Equivalents)), 120% of Vitamin C, and 4% of calcium and iron.

### Data Collection and Measures

Baseline self-reported demographic and dietary characteristics were collected at the start of the study. At weeks 6 and 12, subjects who consumed the juice turned in self-reported questionnaires to indicate their daily consumption of the vegetable juice.

Three-day food records were collected from 2 weekdays and 1 weekend day before study visits at baseline (during the carotenoid washout period), week 6 and week 12. Baseline vegetable intake was not used for statistical analyses; instead the data provided information on low carotenoid diet adherence. The food records were reviewed by a registered dietitian and were entered in duplicate into a database and analyzed with Food Processor software (Version 10.2.0, ESHA research, Inc., Salem, OR). Vegetable servings were quantified according to the DASH diet recommendations. One DASH diet serving of vegetables corresponds to 0.5 cups of MyPyramid vegetables [26] or 4 fluid ounces of vegetable juice [27].

Clinical measurements included blood pressure, weight, height, and waist circumference. Blood pressure measurements were the average of 2 measurements and were taken using an automated system (Vital Signs 300, Welch Allyn Protocol Inc., Beaverton, OR) after the subject was seated for 5 minutes. Mean arterial pressure (MAP), or average arterial pressure during a single cardiac cycle, was calculated using the formula: MAP = [((systolic - diastolic)/3) + diastolic]. Normal MAP range is 70-110. For weight and height measurements, subjects were fully dressed, with the exception that their shoes were removed. Height was recorded on their first visit only using a wall-mounted stadiometer (Ayrton S-100, Prior Lake, MN). Weight was recorded every visit using an electronic scale (Scale-tronix Inc., White Plains. NY). At the baseline visit and at week 12, 24-hour urine samples were collected. The 24-hour urine collection period started immediately after consumption of the assigned vegetable juice treatment and ended 24 hours later. Urine was stored during the 24 hours in insulated bags with ice packs. At baseline, week 6 and 12, blood samples were drawn for comprehensive metabolic panel (chemistry, liver, fasting blood glucose), high sensitivity C-reactive protein, and lipid panel. High sensitivity C-reactive protein, lipid and comprehensive metabolic panels and 24-hour urine analysis were performed by the Clinical Pathology Laboratory of the University of California, Davis Medical Center.

Given previous reports on the potential effects of vegetable intakes on the antioxidant status of subjects [28,29], we measured plasma thiobarbituric acid reactive substances (TBARS) as a marker for oxidative stress; TBARS were determined as previously described [30].

### Statistical Analyses

Data were analyzed for the 86 subjects who completed the 12-week study. The vegetable intake data are for the 80 subjects who completed their diet records at both 6 and 12 weeks. For continuous variables, analysis of variance (ANOVA) and Fisher's PLSD (Statview 5.0.1, SAS Institute Inc., Carey, NC) were employed using 3 groups (0, 8 and 16 fluid ounces of vegetable juice daily) or 2 groups (daily vegetable juice consumption or no vegetable juice consumption). Analysis for categorical variables was conducted using the Chi square test to compare the two exposures (vegetable juice or not). Blood pressure data were analyzed using repeated measures ANOVA with Tukey's post hoc for multiple comparisons or Friedman's repeated measures ANOVA where appropriate (Sigma Stat 3.5, Systat Software Inc, San Jose, CA). Subjects were stratified into normal and pre-hypertensive groups (diastolic blood pressure > 80 mm Hg and/or systolic blood pressure > 120 mmHg) and subjects consuming 8 or 16 fluid ounces of vegetable juice were grouped together to increase statistical power. For all statistical analyses, a p-value < 0.05 was considered statistically significant. Data are presented as mean ± standard deviation.

## Results

**Figure 1:**
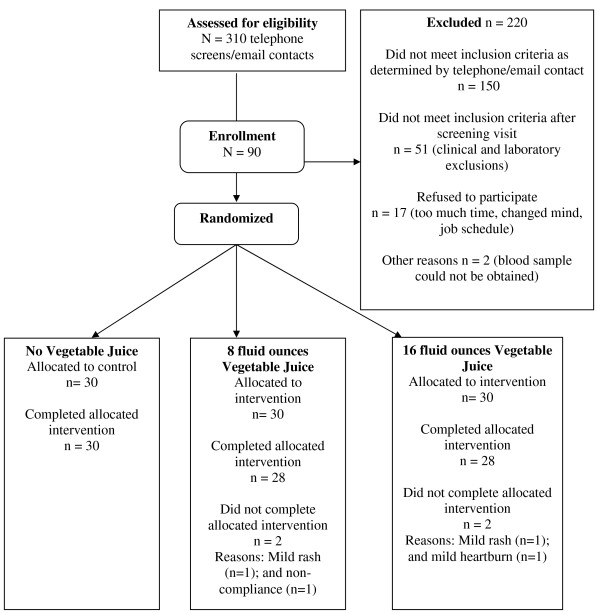
**Enrollment and Randomization**.

A total of 90 subjects were enrolled and randomized to one of three treatment groups. Thirty subjects were assigned to drink 8 fluid ounces of the vegetable juice, 30 subjects were assigned to drink 16 fluid ounces of the vegetable juice, and 30 subjects received no vegetable juice. Of the 90 enrolled, 86 subjects completed the 12-week study. The four subjects who did not complete the study were all within the vegetable juice treatment groups. Two subjects withdrew from the study due to the development of a mild rash, and one subject withdrew due to mild heartburn that they associated with the consumption of the juice. The data for one subject was excluded due to non-compliance (Figure 1). A total of 48 out of 53 participants, who completed beverage consumption logs, indicated that they consistently consumed the juice (≥ 6 days per week of juice consumption), representing a high level of adherence to the protocol. Dietary intake data from six subjects were not analyzed because they had incomplete food records. A total of 80 subjects were included in the analysis of dietary data (n = 27, 26, 27 respectively for 0, 8, and 16 fluid ounces).

Apart from systolic blood pressure and insulin levels, no significant differences in baseline characteristics were observed among the randomized groups. As a matter of chance, baseline systolic blood pressure was significantly higher in subjects randomly assigned to the 8 fluid ounces group than in the other groups. Baseline insulin levels were significantly higher in subjects who were in the 16 fluid ounces group (Table 1). A demographic characteristic of note is the education level of participants; all had completed high school or higher education. Seventy-three percent were college graduates and, of these, 62% had master's, doctoral, or law degrees.

**Table 1:** Baseline Characteristics of the Study Population who Completed 3 Day Diet Records at 6 and 12 weeks

	No beverage (n = 27)	8 fluid ounce group (n = 26)	16 fluid ounce group (n = 27)
Age (years)	51.7 ± 5.1	52.6 ± 7.5	51.0 ± 5.8
Gender, F/M	15/12	21/5	22/5
Weight (kg)	73.8 ± 12.0	70.8 ± 15.7	75.1 ± 13.2
Height (cm)	170.4 ± 7.8	166.9 ± 9.1	168.5 ± 8.1
BMI (kg/m^2^)	25.7 ± 3.2	25.0 ± 4.0	26.5 ± 3.2
Waist Circumference (cm)	88.8 ± 10.6	85.6 ± 9.3	89.8 ± 9.8
Systolic BP (mm Hg)	113.8 ± 8.7	118.7 ± 11.9*	111.5 ± 9.5
Diastolic BP (mm Hg)	73.8 ± 7.2	75.7 ± 8.4	72.9 ± 7.2
Total Cholesterol^∂ ^(mg/dL)	208.3 ± 43.8	213.5 ± 43.4	200.7 ± 37.5
HDL^∂ ^(mg/dL)	51.8 ± 16.4	55.2 ± 15.5	49.3 ± 15.5
LDL^∂ ^(mg/dL)	138.3 ± 41.2	134.9 ± 51.0	126.0 ± 29.3
Chol: HDL	4.5 ± 2.0	4.3 ± 1.7	4.5 ± 1.5
Triglycerides^∫ ^(mg/dL)	101.5 ± 52.9	117.2 ± 85.6	126.8 ± 73.3
hsCRP (mg/L)	1.6 ± 1.9	1.6 ± 1.6	2.0 ± 1.7
Insulin^∩ ^(uU/mL)	6.5 ± 3.7	5.6 ± 2.4	9.0 ± 6.2**
Fasting Blood Glucose^⌂ ^(mg/dL)	92.9 ± 7.4	92.7 ± 8.8	93.4 ± 5.3

Values are mean ± standard deviation.

* Significantly different from 16 ounce group *p *= 0.02; **significantly different from 8 ounce group (*p *= 0.01), and no beverage (*p *= 0.04)

BMI = Body Mass Index; BP = Blood Pressure; Chol = Cholesterol; HDL = High Density Lipoprotein; hsCRP = high sensitivity C-reactive protein; LDL = Low Density Lipoprotein

**Figure 2:**
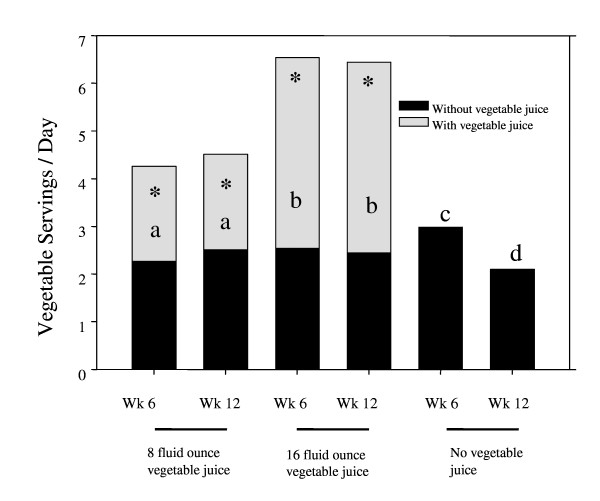
**Comparison of vegetable intake (servings per day) among vegetable juice consumption groups at weeks 6 and 12**. * **a **Significantly different from no vegetable juice **c **at 6 weeks and **d **at 12 weeks *p *≤ 0.01, **b **significantly different from 8 fluid ounces and no vegetable juice groups at 6 weeks, and at 12 weeks *p *≤ 0.0001. Vegetable consumption in the week 12 no vegetable juice group was significantly decreased from week 6 *p *≤ 0.01. 8 fluid ounce group n = 26; 16 fluid ounce group n = 27; no vegetable juice n = 27.

Despite being counseled on the DASH diet and being encouraged to increase their consumption of vegetables, apart from vegetable juice consumption, the majority of people enrolled in the study had a vegetable intake that was lower than public health recommendations, of at least 4 daily servings, or 2 cups, of vegetables (Figure 2). Average vegetable intake for all groups, without counting vegetable juice, was 2.6 servings per day after 6 weeks and 2.3 servings per day after 12 weeks. There were no significant differences among groups in dietary vegetable intake at 6 or 12 weeks (apart from vegetable juice consumption). These intakes are well short of DASH recommendations of 4-6 servings/day for the subjects' calorie requirements of between 1600-3000 calories per day. Adding 8 fluid ounces (2 DASH diet servings of vegetables) of vegetable juice to the diet increased the average daily vegetable servings to 4.3 after both 6 and 12 weeks. The addition of 16 fluid ounces (4 DASH diet servings) of vegetable juice to the diet resulted in an average daily vegetable consumption that met the minimum recommendation for vegetable intake (6.5 and 6.4 servings per day at weeks 6 and 12, respectively). These results were in marked contrast compared to individuals who received only DASH education. Their vegetable intake averaged 3 servings per day at week 6, and 2.1 servings per day at week 12. Apart from vegetable juice consumption, only 7.7% of subjects in the 8 fluid ounces and 18.5% in the 16 fluid ounces group met daily vegetable intake recommendations at week 6. At week 12, 19.2% in the 8 fluid ounces group and 11.1% in the 16 fluid ounces group met the recommendations. In contrast, when vegetable juice was incorporated into the diet, 50% of participants in the 8 fluid ounces group and 100% of those consuming 16 fluid ounces of vegetable juice achieved daily vegetable recommendations at 6 weeks. At week 12, 53.8% in the 8 fluid ounces group and 100% of those in the 16 fluid ounces group met recommendations. For those in the control group, who received only DASH diet instruction and consumed no vegetable juice, 22.2% at 6 weeks and 7.4% at 12 weeks achieved daily vegetable intake recommendations.

The addition of the vegetable juice added vegetable servings to the diet and significantly increased dietary potassium and sodium intake at 6 and 12 weeks in those consuming 16 fluid ounces daily (Table 2). These additions coincided with corresponding increases in the concentrations of urinary sodium and potassium excreted by those consuming the juice (Table 3). Consuming vegetable juice resulted in a dose response increase in dietary vitamin C at 6 and 12 weeks among groups (Table 2) and those consuming 16 fluid ounces of vegetable juice had higher dietary vitamin A than the control group at 12 weeks. This increase in vitamin A came in the form of β-carotene present in the juice. There were no changes in nutrient composition in the reported dietary intakes in any group between 6 and 12 weeks (Table 2).

**Table 2:** Summary of Dietary Data from All Subjects Who Completed 3 Day Diet Records at 6 and 12 Weeks

	Week 6			Week 12		
	No vegetable juice (n = 27)	8 fluid ounce group (n = 26)	16 fluid ounce group (n = 27)	No vegetable juice (n = 27)	8 fluid ounce group (n = 26)	16 fluid ounce group (n = 27)
Calories (Kcal)	1774^ab ^± 518	1637^a ^± 383	2011^b ^± 664	1917^ab ^± 530	1640^a ^± 538	2042^b ^± 564

Without Juice	1774^ab ^± 518	1587^a ^± 383	1961^b ^± 664	1917 ± 530	1590^a ^± 538	1942 ± 564

Protein (g)	77^ab ^± 27	69^a ^± 18	86^b ^± 23	83 ± 27	73 ± 23	83 ± 24

Without Juice	77^ab ^± 27	66^a ^± 18	82^b ^± 23	83 ± 27	70 ± 23	79 ± 24

Carbohydrate (g)	226 ± 65	215 ± 53	249 ± 84	238^ab ^± 66	210^a ^± 75	262^b ^± 82

Without Juice	226 ± 65	204 ± 53	229 ± 84	238 ± 66	200 ± 75	242 ± 82

Fiber (g)	22 ± 9	22 ± 9	26 ± 10	23 ± 10	22 ± 11	27 ± 10

Without Juice	22 ± 9	20 ± 9	22 ± 10	23 ± 10	20 ± 11	22 ± 10

Fat (g)	59^a ^± 27	58^a ^± 26	79^b ^± 46	65^ab ^± 28	56^a ^± 24	72^b ^± 3

Without Juice	59^a ^± 27	58^a ^± 26	79^b ^± 46	65^ab ^± 28	56^a ^± 24	72^b ^± 3

Vitamin A (IU)	8185 ± 4938	7994 ± 4416	12537 ± 1392	7171^b ^± 4531	11206^ab ^± 7237	14568^a ^± 21648

Without Juice	8185 ± 4398	5994 ± 4416	8536 ± 1392	7171 ± 4531	9206 ± 7237	10568 ± 21648

Vitamin C (mg)	106^c ^± 63	148^a ^± 53	232^b ^± 68	95^c ^± 64	173^a ^± 74	230^b ^± 52

Without Juice	106 ± 63	75 ± 53	88 ± 68	95 ± 64	101 ± 74	86 ± 51

Calcium (mg)	789 ± 392	751 ± 355	770 ± 278	880 ± 395	805 ± 364	790 ± 319

Without Juice	789 ± 392	711 ± 355	690 ± 278	880 ± 397	765 ± 364	711 ± 319

Iron (mg)	14^a ^± 4.6	14^a ^± 3.5	18^b ^± 6.1	13^a ^± 4.5	14^a ^± 4.7	18^a ^± 6.9

Without Juice	14 ± 4.6	12 ± 3.5	15 ± 6.1	13 ± 4.5	12 ± 4.7	15 ± 6.9

Potassium (mg)	2080^a ^± 777	2319^a ^± 837	3126^b ^± 837	2252^a ^± 685	2412^a ^± 788	3107^b ^± 887

Without Juice	2080 ± 777	1849 ± 837	2187 ± 837	2252 ± 685	1942 ± 788	2167 ± 887

Sodium (mg)	2447^a ^± 931	2600^a ^± 912	3612^b ^± 1231	2504^a ^± 1134	2841^a ^± 1118	3225^b ^± 953

Without Juice	2447 ± 931	2120 ± 912	2651 ± 1230	2504 ± 1134	2360 ± 1118	2665 ± 953

*Means not sharing superscripts at any single time (6 or 12 weeks) point are significantly different from each other (*p *< 0.05). Values are presented as means ± SD.

**Table 3:** Urinary Sodium and Potassium Concentrations (mEq/L) of the Study Population

	No vegetable juice (n = 24)	8 fluid ounce group (n = 23)	16 fluid ounce group (n = 26)
Urinary Potassium Concentration--0 weeks	21.6 ± 8.1	27.6 ± 15.8	30.2 ± 11.1*

Urinary Sodium Concentration--0 weeks	47.9 ± 21.1	62.8 ± 33.5	71.0 ± 24.8**

Urinary Potassium Concentration--12 weeks	24.9 ± 10.3	28.4 ± 13.0	36.2 ± 16.8***

Urinary Sodium Concentration--12 weeks	58.2 ± 20.8	62.4 ± 23.5	79.0 ± 27.8****

*Significantly different from no vegetable juice group (*p *= 0.01); **significantly different from no vegetable juice group (*p *< 0.01); ***significantly different from no vegetable juice group *p *< 0.01; ****significantly different from 8 fluid ounce group (*p *= 0.02), and no vegetable juice group (*p *< 0.01)

Values are means ± SD.

**Figure 3:**
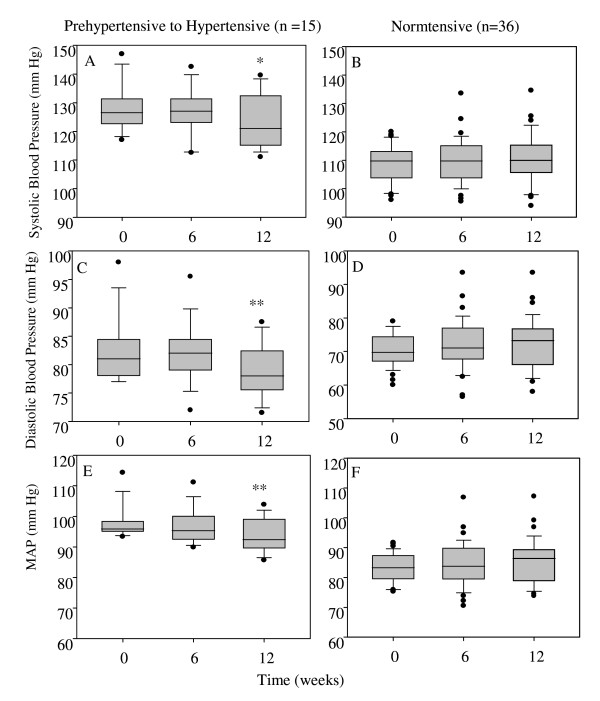
**Twelve Weeks of Vegetable Juice Consumption Decreases Blood Pressure in Individuals with Diastolic Blood Pressure > 80 mm Hg and/or Systolic Blood Pressure > 120 mmHg**. Subjects consuming 8 or 16 fluid ounces of vegetable juice were combined into a single group to examine systolic (A), diastolic (C) and mean arterial pressures (E). Systolic (A and B), diastolic (C and D), and mean arterial (MAP; E and F) pressure of subjects who consumed either 8 or 16 fluid ounces of vegetable juice for 12 weeks. Those who started the study with blood pressures greater than 120 mmHg systolic (A) and/or 80 mmHg diastolic (C) demonstrated a significant decrease in blood pressure over the 12-week period, including MAP (E). Those who had normal pressures at the start of the study demonstrated no significant change in blood pressure over the 12-week period (B, C and F). * Significant from baseline *p *< 0.05 (Repeated Measures ANOVA, Tukey post hoc). ** Significant from baseline, and 6 weeks (*p *< 0.05; Repeated Measures ANOVA, Tukey post hoc).

There were no significant differences in systolic or diastolic blood pressure within groups or among all treatment groups, when all subjects were included, over the 12-week period. However, significant differences in blood pressure were observed in a subset of individuals who drank the juice. Figure 3 compares those individuals who drank the juice for 12 weeks (8 and 16 fluid ounces data combined), and had blood pressures at the start of the study over 120 mm Hg systolic or 80 diastolic (panels A, C, E) with those who had normal blood pressure (panels B, D, F). Those who began the study with normal blood pressures had no significant change in blood pressure throughout the study. In those subjects who started the study with blood pressure in the pre-hypertensive range, a significant within group decrease in all blood pressure parameters was observed after 12 weeks of juice consumption. Systolic blood pressure significantly decreased after 12 weeks of juice consumption (128.1 ± 8.0 at baseline vs. 123.4 ± 9.5 at week 12; Figure 3A; *p *< 0.05). After 12 weeks of vegetable juice consumption, diastolic blood pressure was significantly reduced from baseline and 6 weeks (82.6 ± 5.6 at baseline and 81.8 ± 5.2 at week 6 compared to 79.0 ± 4.5 at week 12; Figure 3C; *p *< 0.05). Similarly, mean arterial pressure was significantly reduced after 12 weeks of juice intake from both baseline and at 6 weeks (97.8 ± 5.3 at baseline, 96.8 ± 5.7 at week 6, and 93.8 ± 5.4 at week 12; Figure 3E; *p *< 0.05).

There were no significant differences within groups over the 12 week intervention, or among groups, in BMI, waist circumference, plasma insulin, measures from the comprehensive metabolic panel (chemistry, fasting blood glucose, and liver panel), lipids, high sensitivity C-reactive protein or plasma TBARS (data not reported).

## Discussion

Relative to current public health recommendations, the majority of Americans consume inadequate amounts of vegetables (minimum of 4 servings) in their diet [7,31,32]. Consistent with this report, our subjects' average daily intake of vegetables did not meet recommendations (for either DASH or 2005 Dietary Guidelines) at any time during this study despite DASH diet education. Importantly, the dietary intake of vegetables apart from the juice was similar among the three groups. However, the incorporation of the vegetable juice significantly increased the total daily intake of vegetables to an average of 4.3 and 6.5 servings of vegetables in the 8 and 16 fluid ounce groups, respectively. The DASH diet instruction emphasized inclusion of vegetables, and did not instruct subjects to consume vegetable juice in place of mealtime vegetable servings. These results support the concept that the daily consumption of a vegetable juice is a feasible way for an individual to help meet their dietary recommendation for vegetable intake. During the study we obtained information from participants related to their perceptions on including vegetable juice into their diets. The majority of those drinking the juice self-reported that they enjoyed the taste, felt they were doing something good by drinking it, were satisfied they had a diet that provided the right vitamins and minerals, and were satisfied with the ease of getting vegetables in their diet.

The data from the current study were obtained from a population of healthy, well-educated adults in California. Similar to what is found in various sectors of the U.S. population [33-35], the majority of people enrolled into this study did not consume the recommended daily servings of vegetables. Indeed, reported intakes at the 12-week point in the control group were lower than those reported in these previous national surveys. In a study of African American men, Wolf et al. found that they were not aware of, nor meeting, dietary recommendations due to a lack of knowledge and perceived barriers [35]. Similarly, McGee et al. [14] found that individuals in the lower Mississippi delta felt that they lacked knowledge and skills to prepare meals with healthful food choices. However, our subject population was provided with education on developing meals plans and tips for making healthy eating easier. This dietary education, coupled with the high education level of the individuals enrolled in the current study, should have resulted in their meeting DASH recommendations. Based on our data, we suggest that behavior change for an extended time period, even for highly educated individuals, is difficult. Studies suggest that beneficial changes in behavior for even motivated individuals require intensive, extended behavioral counseling, relapse prevention training, incentives, self efficacy and social support [36,37]. Results from a meta analysis conducted to evaluate the overall effectiveness of behavioral interventions to promote dietary change in fruit and vegetable intake were not conclusive [38].

Our results confirm that dietary training alone is not enough to elicit change; this finding underscores the need for new modalities to encourage beneficial dietary modification. Guenther et al. [31] and Casagrande et al. [7] emphasize the need for broadening interventions beyond increasing individual awareness of the value of vegetables and education toward altering eating behavior. While several studies have shown successful improvements in vegetable consumption through various dietary behavior change programs [37,39], others have not reported these benefits [40,41]. More effective intervention possibilities may include increasing access to vegetables [42]. The need for lower calorie, nutrient dense, convenient [12], inexpensive [43] methods to increase vegetable consumption are important for future public health campaigns. Our data suggests that vegetable juice may serve as a portable and convenient method to aid individuals in meeting dietary vegetable recommendations.

Although vegetable consumption was enhanced in those consuming juice, no significant changes were observed over the 12-week test period for any of the measured vascular health parameters listed in Table 1. The vegetable juice in the study provided a one to one ratio of sodium to potassium and thus it is important to note that blood pressure measurements did not change in any of the groups over the 12 week period. The group blood pressure results obtained in the current study are consistent with previous reports that show total dietary mineral intake is a consideration relative to blood pressure [44-46]. To further probe this issue, we analyzed the data from the subset of individuals whose initial blood pressures were over 120/80 mm Hg (pre-hypertensive but less than hypertensive range). In the vegetable juice group, this subgroup showed a significant decrease in blood pressure over the 12 week intervention period (the number of pre-hypertensive subjects in the no vegetable juice group was too low (N = 7) to allow for meaningful statistical comparisons for this specific subgroup). While preliminary in nature, our finding that the consumption of the vegetable juice was associated with a reduction in blood pressure in pre-hypertensive subjects is consistent with the putative positive vascular health effects of diets rich in vegetables, which are good sources of dietary potassium. This differential blood pressure response between normotensive and pre-hypertensive individuals has been seen in other intervention studies [47,48].

Svendsen et al. [49] reported improvements in blood pressure following an intervention promoting very high levels of vegetable and fruit intake. Those improvements were seen in a more compromised population exhibiting a higher baseline body mass index and included some individuals with cardiovascular risk factors such as hypertension and diabetes mellitus, smokers, and those with a prior cardiovascular event [49].

In several papers, the concept has been raised that vegetables or their respective extracts can result in an improvement in the oxidant defense system, with a consequential reduction in tissue oxidative damage [50-52]. While the measurements are controversial [53], numerous investigators have used plasma TBARS as a general indicator for oxidative damage [54,55]. For the above reason, this measurement was included in the current study. However, consistent with our expectation, the consumption of the vegetable juice product did not result in significant changes in this parameter. We think it is important to note, however, that the above finding does not rule out the possibility that the vegetable juice intervention could have improved select parameters of the oxidative defense system. For example, it is possible that phytochemicals present in vegetable juice could reduce the extent of the oxidation of select targets, such as low density lipoproteins (LDL) [22] and deoxyribonucleic acid (DNA) [56,57]. However, it is likely that these phytochemicals are acting, in part, through mechanisms that are independent of the direct antioxidant properties of the phytochemicals. For example, it has been suggested that certain phytochemicals can inhibit enzymes that produce reactive oxygen and nitrogen species [58,59]. Additional research similar to that conducted by Valtueña et al. [60], correlating the intake of adequate vegetables, containing dietary antioxidants, to functional antioxidant and oxidative stress parameters is needed.

An important consideration for our study is that the subject selection does not reflect the general population because it was a fairly homogenous, highly educated, Caucasian, healthy population with very high adherence and willingness to volunteer for a study that included drinking vegetable juice. The current findings need to be replicated in a larger, more diverse population group, and ideally it needs to be conducted over a longer period of time.

## Conclusion

Our results show that the consumption of vegetables, even in an educated, healthy population, remains lower than recommendations. Registered dietitians can help clients enhance vegetable intake and bridge the vegetable gap by recommending the incorporation of 8 fluid ounces of vegetable juice into their diets. Interestingly, increasing daily vegetable intake through the incorporation of vegetable juice was associated with reduced blood pressure in a subset of pre-hypertensive subjects. Future research should be done to examine other at risk subgroups, including minorities and individuals with pre-existing cardiovascular risk factors to better define the cardiovascular risk reductions that are associated with the chronic consumption of vegetable juice.

## Competing interests

This work was supported in part by resources from the Campbell Soup Company and in part by resources provided from the UC Davis Center for Health and Nutrition Research which was established with funding from the State of California Vitamin Price Fixing Consumer Settlement Fund. CS Khoo and BL Winters are employees of Campbell Soup Company and hold stock there. CL Keen and JP Foreyt are members of Campbell Soup Company's Vegetable Plant Advisory Panel. SF Shenoy, WSC Poston, RS Reeves, AG Kazaks, RR Holt, HJ Chen, CK Haddock and ME Gershwin do not have any financial interests to declare.

## Authors' contributions

SFS acquired and interpreted data, drafted and critically reviewed the manuscript, and gave final approval of the version to be published. WSCP analyzed and interpreted data, drafted and critically reviewed manuscript and gave final approval of the version to be published. RSR interpreted data, drafted and critically reviewed manuscript, and gave final approval of the version to be published. AGK acquired and interpreted data, drafted and critically reviewed the manuscript, and gave final approval of the version to be published. RRH made substantial contributions to conception and design of study, acquired and interpreted data, drafted and critically reviewed the manuscript, and gave final approval of the version to be published. CLK made substantial contributions to conception and design of study, interpreted data, drafted and critically reviewed the manuscript, and gave final approval of the version to be published. HJC acquired data, drafted and critically reviewed the manuscript, and gave final approval of the version to be published. CKH analyzed and interpreted data, drafted and critically reviewed manuscript and gave final approval of the version to be published. BLW made substantial contributions to conception and design of study, interpreted data, drafted and critically reviewed the manuscript, and gave final approval of the version to be published. CSK made substantial contributions to conception and design of study, interpreted data, drafted and critically reviewed the manuscript, and gave final approval of the version to be published. JPF made substantial contributions to conception and design of study, interpreted data, drafted and critically reviewed the manuscript, and gave final approval of the version to be published. MEG interpreted data, critically reviewed manuscript and gave final approval of the version to be published.
